# IMplementation and evaluation of the school-based family support PRogram a Healthy School Start to promote child health and prevent OVErweight and obesity (IMPROVE) – study protocol for a cluster-randomized trial

**DOI:** 10.1186/s12889-021-11663-2

**Published:** 2021-09-06

**Authors:** Liselotte Schäfer Elinder, Camilla A. Wiklund, Åsa Norman, Nouha Saleh Stattin, Susanne Andermo, Emma Patterson, Erik Hemmingsson, Clayton Cook, Sara Raposo, Lydia Kwak

**Affiliations:** 1 Department of Global Public Health, Karolinska Institutet, SE-171 77 Stockholm, Sweden; 2 Centre for Epidemiology and Community Medicine, Region Stockholm, SE-104 31 Stockholm, Sweden; 3 Department of Clinical Neurosciences, Karolinska Institutet, SE-171 65 Solna, Sweden; 4 Academic Primary Healthcare Centre, Region Stockholm, SE-113 65 Stockholm, Sweden; 5 Department of Neurobiology, Care Sciences and Society, Karolinska Institutet, SE-141 83 Huddinge, Sweden; 6 Department of Physical Activity and Health, Swedish School of Sport and Health Sciences, SE-114 68 Stockholm, Sweden; 7 Department of Organizational Leadership and Policy Development, University of Minnesota, Minneapolis, MN 55 USA; 8 Institute for Environmental Medicine, Karolinska Institutet, SE-171 77 Stockholm, Sweden

**Keywords:** BMI, Diabetes, Diet, Fidelity, Hybrid type 3 design, Health promotion, Implementation strategies, Physical activity, Primary health care

## Abstract

**Background:**

IMPROVE aims to conduct a hybrid type 3 evaluation design to test the effectiveness of bundled implementation strategies on intervention fidelity of the Healthy School Start (HSS) program, while simultaneously monitoring effects on health outcomes of children and parents. The HSS is a 4-component family support program for children starting school (5–7 years of age) promoting healthy dietary habits and physical activity in the home environment to prevent childhood obesity and parents’ risk of developing type 2 diabetes.

**Methods:**

IMPROVE is a cluster-randomized controlled trial with two arms to evaluate and compare the effects of two different bundles of implementation strategies on intervention fidelity expressed as adherence and responsiveness at 12 and 24 months (primary outcomes). Thirty schools in two municipalities will participate in the study reaching about 1400 families per school year. In stakeholder workshops, key implementation determinants were identified according to the domains of the Consolidated Framework for Implementation Research. Through a consensus process with stakeholders, two bundles of implementation strategies were tailored to address context-specific determinants. Schools randomly assigned to group 1 will receive bundle 1 (Basic) and group 2 will receive bundle 1 + 2 (Enhanced). Bundle 2 consists of external facilitation, fidelity monitoring and feedback strategies. Secondary outcomes will include change in acceptability, appropriateness, feasibility, and organisational readiness as perceived by school staff. In addition, child weight status and diet, and parents’ feeding practices and risk of type 2 diabetes will be monitored. Linear and ordinal regression analysis will be used to test the effect on the primary and secondary outcomes, taking clustering and covariates into consideration where needed. Process evaluation will be conducted through key stakeholder interviews to investigate experiences of the program and perceptions on sustainability.

**Discussion:**

This systematic approach to investigating the effectiveness of two different bundles of implementation strategies tailored to context-specific determinants on the fidelity of the HSS intervention will provide new insight into feasible implementation strategies and external support needed for the HSS to be effective and sustainable. Results will help inform how to bridge the gap between the research on school-based health programs and routine practice in schools.

**Trial registration:**

Registered prospectively at ClinicalTrials.gov ID: NCT04984421, registered July 30, 2021

**Supplementary Information:**

The online version contains supplementary material available at 10.1186/s12889-021-11663-2.

## Contributions to the literature

- Comparing the effectiveness of two bundles of implementation strategies tailored to context specific determinants identified by stakeholders
- Adds to the knowledge on hybrid design studies in implementation research
- Adds new perspectives on the construction of a fidelity score in a complex intervention
- Adds knowledge on the form and function for external facilitation support in the school setting

## Background

An unhealthy diet and lack of physical activity are among the most important risk factors for chronic diseases like cardiovascular diseases, different types of cancers and diabetes in Sweden and globally [1]. In addition, these health behaviours increase the risk of overweight and obesity from early childhood and throughout the life course [2]. More than half of the adult population in most high-income countries now is overweight or obese [3]. Previous studies from Sweden and other high-income countries have shown that children of parents with lower educational attainment [4, 5] and non-Nordic origin [6, 7] have a higher consumption of unhealthy foods and also higher levels of obesity [8, 9] than children of parents with higher educational attainment and who are Swedish-born. There is consistently a 2–3 fold lower prevalence of childhood overweight and obesity in areas with higher socioeconomic status (i.e. income and education) compared to disadvantaged areas [10], which is also found among adults [11]. Given this, there is a need for interventions that start at an early age and target the whole family. Systematic reviews have concluded that due to accessibility to reach nearly all children in a given community, schools are an ideal setting for the delivery of health programs, particularly for children 6–12 years of age [2, 12]. Effective school-based programs are characterised by active parental involvement, especially for younger children [13, 14]. Parents are key in shaping the food and meal environment, providing and reinforcing opportunities for physical activity, model healthy habits, and establish social and cultural norms that promote health for their children [15–17].

### Determinants of implementation and implementation strategies

In order for evidence-based programs to affect population health, they have to be implemented with high fidelity at scale and sustained over time, which has been difficult to achieve [18], especially for more complex multi-level programs. One reason for this is that context-specific determinants of successful implementation are often not identified and used to inform the development of tailored implementation strategies [19]. The Consolidated Framework for Implementation Research (CFIR) is a useful framework that can be used by implementation researchers with community partners to identify context-specific determinants potentially influencing implementation. It includes 39 constructs across five domains; the intervention itself, the inner setting, the outer setting, characteristics of providers, and the implementation process [20]. Three of the 39 constructs have repeatedly been shown to relate to implementation success, namely stakeholders’ perception of the advantage of the intervention relative to other programs, the tension for change as perceived by stakeholders, and if clients’ needs are prioritised by the organisation and resources are present [21]. Other important constructs are relative priority and planning [21]. To accommodate the multi-level nature of interventions including both children and parents, a sixth domain in the CFIR has been proposed called “characteristics of intervention recipients” [21]. A construct that has received increasing attention in the inner setting as an important factor for implementation success is the construct “Readiness for Implementation”, also called organizational readiness [22]. Organizational readiness is a determinant of successful adoption which is necessary for implementers to persist toward fidelity.

Developing implementation strategies that are tailored to context-specific implementation determinants is more likely to improve fidelity to the intervention than no strategies or simple dissemination of guidelines [23]. Determinants of implementation at both individual (provider and service recipient) and organisational levels should therefore be carefully identified as a first step, preferably in collaboration with key stakeholders [24]. However, it is not yet clear how best to tailor strategies and therefore not clear what the magnitude of the effect is that tailored implementation strategies have on both implementation and service recipient outcomes [24]. Assessment of fidelity to the intervention components is one way of evaluating the effectiveness of tailored implementation strategies.

The Expert Recommendations for Implementing Change (ERIC) Project has yielded a significant contribution to establish a common language and taxonomy of 73 unique implementation strategies in health [25]. Cook et al. adapted the ERIC taxonomy for use in the school context, with 75 unique implementation strategies from which to choose and tailor strategies to context-specific barriers, the SISTER taxonomy [26]. A recent study by Lyon et al. including 200 school-based consultants who support social, emotional, and mental health services found that the five strategies rated as most important were: (a) Conduct ongoing training, (b) Make training dynamic, (c) Provide ongoing consultation/coaching, (d) Monitor the progress of the implementation effort, and (e) Improve implementers’ buy-in. The five strategies rated as most feasible were (a) Make training dynamic, (b) Distribute educational materials, (c) Remind school personnel, (d) Facilitation/problem solving, and (e) Capture and share local knowledge. Using these types of implementation strategies in isolation as standalone approaches is not advised as there are numerous determinants influencing successful implementation. Rather, what is needed is a bundle of strategies that target different barriers within a given context to achieve high fidelity to and effectiveness of school-based interventions. Reporting on fidelity to the implementation strategies is equally as important to understand how and why successful implementation of the intervention comes about [27]. Strategies should be clearly described according to features such as a) the actor; b) the action; c) the action target; d) temporality; e) dose; f) implementation outcome affected; and g) the justification for use [28].

### The Healthy School Start program

Development of the Healthy School Start program (HSS) began in 2010 and to date the program has been evaluated in three waves. It is based on Social Cognitive Theory with parental self-efficacy, role modelling, and observational learning as central constructs and mediators of child behaviour change [29]. The program is designed to run in compulsory pre-school class or grade 1 (child age 5–7 years) and is universal (reaches all children). It can be characterised as a structured work routine to fulfil the requirements in the curriculum for teachers to teach about health. It is also fully in line with the Swedish national guidelines for school health care concerning health promotion and prevention of overweight and obesity [30]. Despite the existence of these guidelines, no specific evidence-based practice is recommended. This results in inconsistencies in practice and negative parental responses are often encountered by school nurses when it comes to the issue of child weight development [31].

The program targets parents of children during a developmental period in which health-related behaviours are still very much under the influence of parents [29]. The program is delivered by teachers, school nurses and primary health care staff, and involves four core components: 1) A health information brochure to parents; 2) Motivational interviewing (MI) sessions with parents performed by the school nurse; 3) Classroom activities performed by teachers with home assignments to be completed by children together with their parents; and 4) a web-based self-test of type 2 diabetes (T2D) risk for parents with a recommendation to seek medical advice in primary health care, if needed. These four components were designed to be complementary and mutually reinforcing as emphasised by Gittelsohn [32], who summarised challenges and lessons learnt from multi-level and multi-component interventions to prevent and reduce childhood obesity in different countries. In previous trials of the HSS program, the following implementation strategies used were used based on experience and the literature: 1) Obtain formal commitments, a written agreement with each school specifying school and researcher obligations; 2) Distribution of educational materials like the health brochure, information on the project website and video instructions for teachers; 3) Ongoing training, MI training for school nurses; and 4) Prepare families to be active participants through a kick-off meeting for school staff and parents together.

The program has been evaluated in schools with mixed socioeconomy [33] and in disadvantaged areas [34] in the greater Stockholm area. Fidelity to the intervention components was high for the MI component performed by external MI counsellors in the first two trials, and the classroom component performed by teachers. In the first trial [33] we found significant beneficial effects on dietary habits (higher consumption of fruit and vegetables and lower consumption of unhealthy foods and drinks in the intervention group relative to control). In the second trial [34] we also found a significantly lower BMI z-score in children with obesity at baseline compared to control. In an attempt to increase the involvement of parents in the program, a validated short non-invasive tool to identify individuals at high risk for type 2 diabetes (FINDRISC) [35] was added to the third trial. Undiagnosed diabetes and prediabetes are common in Sweden [36]. If detected early, lifestyle modification can reverse this condition [37] and potentially have beneficial effects on the whole family. In addition, an increased emphasis was placed on parental feeding practices and child physical activity, and the MI was conducted by trained school nurses in the third trial [29]. Results from the third trial running from 2017 to 2019 are in line with the previous studies (manuscript submitted).

During previous evaluations of the HSS program the following implementation barriers were encountered: low priority given to health promotion in schools relative to achievement of learning outcomes [38], lack of positive leadership [39], lack of preparation time [40], the need for clear communication and collaboration between teachers and parents, especially regarding the children’s home assignments [41], and difficulty to engage parents in low-income areas due to stressful everyday life and lack of knowledge and parenting skills [16, 39]. Furthermore, it was found that commitment among staff is important for high intervention fidelity [38]. Addressing these barriers could be achieved through the establishment of an implementation team [38], and by receiving resources and support from external partners such as public health teams [42, 43]. Ultimately, there are numerous barriers that impact the successful implementation of school-based health programs like the HSS that warrant tailored approaches that target these determinants to achieve high fidelity.

The overarching purpose of IMPROVE is to conduct a hybrid type 3 evaluation design to test the effects of bundled implementation strategies on the intervention fidelity of the HSS program while simultaneously monitoring effects on health outcomes of children and parents. There are two primary aims of IMPROVE: 1) To assess the comparative effectiveness of two bundles of implementation strategies (Basic and Enhanced) on the fidelity to the HSS program components; and 2) to monitor effects of the HSS program on child (diet and weight status) and parent outcomes (feeding practices and T2D risk). This study protocol follows the SPIRIT guideline [44] (Additional file 1).

### Specific aims and hypothesis

The hypothesis is that the use of tailored implementation strategies with a focus on facilitation, monitoring, and feedback will lead to significantly better fidelity to the HSS intervention and to sustained effects on child and parent health outcomes. This study will be guided by the following research questions (question 1 has already been addressed):

1. What context-specific implementation determinants do stakeholders from municipalities in Sweden identify with regard to the implementation of the HSS program?
2. Which of two bundles of tailored implementation strategies is most effective at improving fidelity, acceptability, appropriateness, feasibility, and organisational readiness to implement the HSS program?
3. Does implementation of the HSS program at scale reduce social disparities in the proportion of children with overweight and obesity, and T2D risk among adults?
4. What external implementation support will be needed to facilitate the sustainment of the program after the end of the study?

## Methods

### Study design

**Fig. 1:**
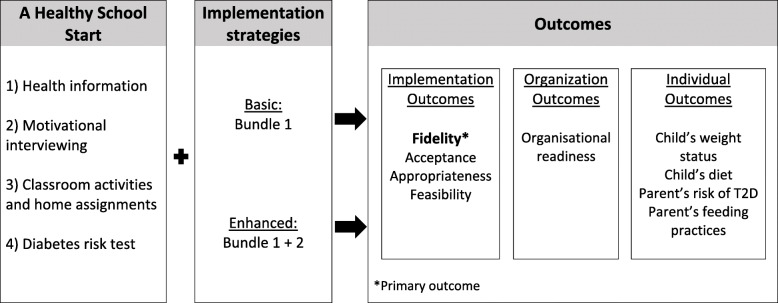
Program theory of IMPROVE

A hybrid type 3 cluster-randomized implementation effectiveness trial with 2 parallel arms (Basic and Enhanced) will be conducted (Fig. 1). Hybrid type 3 trials experimentally test the effects of implementation strategies on implementation outcomes while also gathering data on the effects on service recipient outcomes [45]. The primary dependent variable will be fidelity to the HSS (implementation outcome). Using a hybrid type 3 design is advantageous when (a) there is a high momentum and mandate for implementation despite a somewhat limited evidence base, (b) effects are suspected to be vulnerable to a “voltage drop” when scaling up into routine practice, and c) the implementation strategies being tested are reasonably feasible in context [45]. Figure 1 shows the program theory of IMPROVE using the evaluation model by Proctor et al. [46].

### Setting of the study

Eight municipalities in the Stockholm Region, Sweden, which had previously shown interest in the HSS program were contacted during the fall of 2020 to inquire about their willingness to take part in the IMPROVE study. Information meetings were held between the research team and key personnel such as central administrators and coordinators of school health care to create interest by explaining the rationale and background to the HSS. Two municipalities finally accepted this invitation after discussing it with their education administration. These municipalities had 110,000 and 50,000 inhabitants respectively, with mixed socioeconomy.

### Implementation strategies used during the planning stage

Implementation strategies to be performed by the research team and school staff are named in this protocol according to the SISTER taxonomy specific for the school context [26]. Expert implementation support was offered as well as free training of school nurses in MI (at a value of ca. 1000 Euro per nurse) to incentivise participation in the study (implementation strategy 51: Improve implementer’s buy-in). The participating municipalities signed a memorandum of understanding with the research team that acknowledges their commitment to the study, and describes the role of each partner (strategy 31: Obtain formal commitments). All public primary schools in the municipalities with preschool classes were invited to participate in the study (*n* = 38). Online information meetings with all groups of involved school personnel were held in the spring of 2021 during which they were introduced by the research team to the HSS and the IMPROVE study and given the opportunity to ask questions. After this, school principals were formally asked if their school wanted to participate, which eight of them declined, mainly due to issues related to the covid-19 pandemic. Before the start of the study all involved school personnel will be asked to consent to be included in the study.

### Eligibility of families, inclusion criteria

All children starting pre-school class (5–7 years of age) during the school year 2021/22 and 2022/23 and their parents/guardians are eligible for the study. The HSS program will be presented to parents as part of ordinary school routines, which means that all children and parents will take part in the program and be invited to participate in the study. At the first meeting in school with the class teacher, parents will receive oral and written information about the HSS and the IMPROVE study and a consent form. They will be informed that all families will be exposed to the HSS as part of school routines, but that participation in the IMPROVE study is voluntary and that no personal information or identities will be disclosed.

### Randomisation and blinding

**Fig. 2:**
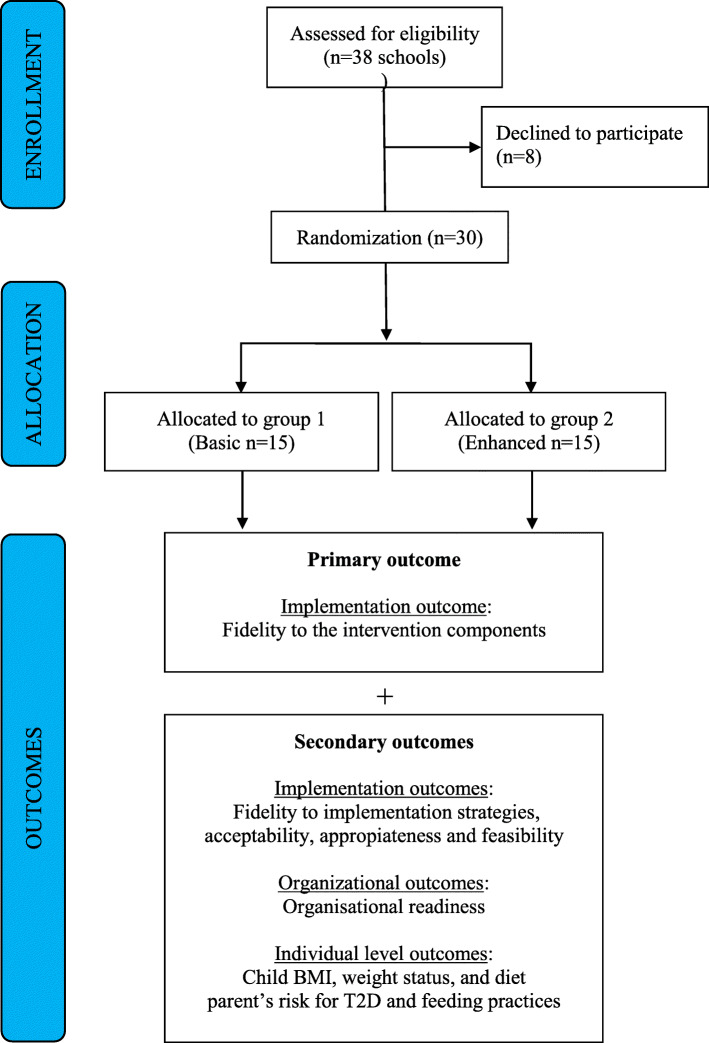
The CONSORT flow diagram of the IMPROVE study

Within each municipality, schools will be randomised 1:1 to group 1 (Basic: bundle 1) or group 2 (Enhanced: bundle 1 + 2) using a computer-generated assignment by an independent statistician after baseline measurement has been conducted in September 2021. It is not possible to blind school staff and the research team to allocation, but parents and children will not get this information. The CONSORT flow diagram with the number of schools and children is shown in Fig. 2.

### The Healthy School Start program

The HSS intervention is a universal intervention designed for all children in pre-school class over the course of one school year. It includes four intervention components described in the previous HSS study protocol [29] and briefly below.

#### Component 1: Health information

At the beginning of the school year parents will receive a brochure with health information based on the Swedish guideline for diet and physical activity [47]. The focus of the brochure is on evidence-based advice concerning parenting practices in relation to healthy food and family mealtimes, sweets, snacks, ice-cream, soft drinks, marketing of unhealthy products, fruit and vegetables, physical activity, sedentary behaviour, screen time, sleep, and cooperation between parents. The brochure is available in Swedish, English, Arabic and Somali and can also be downloaded from the project website.

#### Component 2: Motivational interviewing

MI is a client-centred, directive method for enhancing intrinsic motivation to behaviour change [48]. MI is today regarded as an evidence-based counselling method for behaviour change regarding diet and physical activity in adults [49] and shows promising results in parental support interventions on child health-related behaviours as well [50]. The MI session with the parents will be scheduled by the school nurse as part of the ordinary health visit of children in pre-school class. Parents will be offered a face-to-face MI by the school nurse conducted without the presence of the child and lasting for approximately 20 to 30 min. If special needs are encountered, a follow-up visit will be offered.

#### Component 3: Classroom activities with home assignments

Nine classroom sessions of approximately 30 min duration will be delivered by the teacher in preschool class based on a teacher’s manual developed for this program. In addition, a pedagogic workbook for children with home assignments to be performed together with parents will be handed out to each child. Teachers will receive video-recorded instructions on how to use the teacher’s manual and parents receive instructions and tips on how to practice positive parenting while doing the homework together with their child.

#### Component 4: Type 2 diabetes risk test online

This test contains eight questions and can be performed on paper in the health information brochure and/or online. FINDRISC is a validated tool used to identify individuals at high risk of developing T2D in the coming 10 years [51]. Questions included concern age, BMI, waist circumference, level of physical activity, consumption of vegetables, fruits and berries, history of antihypertensive medication, history of high glucose values and family history of diabetes. In case of medium or higher risk (≥12 points), the parent will be recommended to contact their primary health care centre to confirm prediabetes or T2D according to standard procedures. If the person has T2D or prediabetes then he/she will receive a general check-up, health behaviour counselling and appropriate treatment with yearly follow-up. All primary health care centres in the two municipalities will receive a yearly information letter explaining the intervention and the study.

### Standard practice in Swedish school health care

The national guidelines for school health care are formulated in general terms [30]. They require that the staff engages in health promotion and disease prevention activities and provide support to children in reaching educational goals. No referral is made to specific evidence-based procedures or practices. All school children are offered at least three visits to the school health care between pre-school class (age 6) to grade 9 (age 15). During the first visit to the school nurse in pre-school class the child is accompanied by parents or guardians for a discussion regarding the school situation, the child’s overall well-being, relations with friends, possible health and learning problems, dietary habits and physical activity. The child’s height and weight is measured and compared to standardised growth curves. Eyesight and hearing are also tested. If a child is overweight, additional counselling should be offered including a follow-up. In case of obesity, the school doctor should become involved, and if necessary, the child has to be referred to medical specialists in child obesity for treatment in dialogue with school health care and parents.

### Development of IMPROVE

Development of the IMPROVE study was done in four steps from September 2020 to June 2021 in collaboration with stakeholders in the municipalities and guided by the implementation strategies in the SISTER taxonomy [26].

#### Step 1: Exploring barriers and facilitators of implementation

Exploration of context-specific implementation barriers and facilitators was based on previous process evaluations of the HSS program [16, 38, 39, 41] and on two preparatory stakeholder workshops in each of the two municipalities participating in IMPROVE (Implementation strategy 1: Assess for readiness and identify barriers and facilitators). The CFIR framework was used as a guiding determinant framework due to its multilevel structure and its applicability to the municipality setting [20]. The workshops were held digitally in each municipality due to the Covid-19 pandemic prohibiting in-person workshops. Workshop 1 for municipality 1 was held in November, 2020, with 17 participants (4 from the research team, 2 from primary health care, 2 school principals, 5 school nurses, 3 teachers, and 1 central manager) and lasted for 2.5 h. Workshop 1 for municipality 2 was held in January, 2021, with 18 participants (4 from the research team, 3 from primary health care, 4 school nurses, 1 teacher, 2 school principals, 2 meal staff, and 2 managers) and also lasted for 2.5 h. Each workshop started with the research team presenting the HSS program and the purpose of the IMPROVE study and participants could ask questions for clarification. Then, a brainstorming session was conducted using a virtual white board where participants individually or in small groups for 30 min could contribute “notes” on perceived implementation determinants of HSS in their municipality within five categories: the school, primary health care, the home, the municipality, and “other areas”. In total 42 notes were submitted in municipality 1 of which 15 were marked as a barrier, 5 as both a barrier and a facilitator and 22 as facilitators. In municipality 2, 35 notes were submitted of which 13 were marked as barriers and 22 as facilitators.

After the first round of workshops, the research team coded the barriers and facilitators and grouped them according to the domains of CFIR. Two domains were added to the original CFIR framework: “Characteristics of intervention recipients” as already suggested by Barwick et al. [21] and “Inner setting primary care”. The determinants of intervention recipients (parents) were identified based on the experience of school personnel and confirmed by information from previous qualitative studies with parents participating in the program [16, 39, 40, 52]. The barriers and facilitators were then condensed by the research team into a list of more value-neutral implementation determinants in a consensus-based process. The determinants identified in the workshops were checked against previous process evaluations of the HSS intervention. In total 28 determinants were identified (Table 1) and exemplified by notes made during the workshops by the participants.

**Table 1:** Implementation determinants identified by stakeholders

CFIR domain	Determinants of implementation	Example of notes made by workshop participants
Intervention characteristics (HSS)	1. Program supported by guidelines and curriculum	*“The content/purpose can be linked to the curriculum”*
	2. Involvement of parents	*“Children and parents can get support for a healthier lifestyle and parents can get help with strategies to reach them. Good with MI for self-motivation”*
	3. Consensus on healthy lifestyle	*“Very good with increased and equal knowledge to students and parents about diet, exercise, and health, regardless of background and resources*”
	4. Clarity of material and instructions for use	*“Get a teaching plan for an important part of the student’s development towards a healthy life”*
Outer setting (municipality)	5. Societal action and cooperation	*“That these social institutions work together on the health of the population. Can increase understanding of each other’s mission but also that we share the health mission to reduce social health inequalities”*
	6. Environmental factors	*“In disadvantaged settings, it can be an economic issue, fruits and vegetables are relatively expensive, junk food cheaper, parents can’t afford to let children practice sports”*
	7. Local policies	*“Link to activities and policies that are decided upon”*
Inner setting #1 (school)	8. Common goals and collaboration in school	*“That the school works towards common goals, school health care and teachers work with health promotion and start from the same point of departure and “language” when talking to children and parents”*
	9. Communication and collaboration between school-home	*“Good to have increased consensus between school staff and parents regarding good nutrition and practices around this”*
	10. Continuity in staffing and work routines	*“That there may be staff changes that make implementation difficult both within school health care and teachers”*
	11. Leadership in school	*“Important that all principals endorse the project at their school”*
	12. Visible priority of health	*“That the school works towards a common goal and is consistent in its communication with parents”*
Inner setting #2 (primary care)	13. Communication within and between primary health care units	*“It will be a challenge to communicate to all primary health care centres and to all its individual employees about what the project is about and what help should be offered to those who contact them”*
	14. Time for administration in primary health care	*“Fear that there will be a lot of administrative work for the caregivers. Important to avoid”*
	15. Collaboration between primary care and school	*“Proposal to address IMPROVE at an annual meeting between primary care and school health care”*
	16. New target groups	*“At the primary health care centre you can find other health risks in the parents if they have high blood sugar”*
	17. Early prevention	*“A good opportunity for primary care to work more with prevention and to reach risk groups in time”*
Intervention recipients (parents)	18. Family consensus on healthy lifestyle	*“Very good with increased and equivalent knowledge to students and parents about diet, exercise and health regardless of background and preconditions”*
	19. Parents’ knowledge and skills	*“Parents are included in their children’s homework and together they learn about good habits. Teachers follow up and remind about the homework”*
	20. Socioeconomic and cultural factors, language	*“Socio-economically weak areas - risk of lower adherence to homework being completed - high risk of attrition”*
	21. Parents’ perceived need and readiness	*“Children with overweight/obesity often have parents with the same problems. Difficult to motivate for lifestyle changes”*
	22. Parents’ engagement in children’s schoolwork	*“Poor adherence to homework. The resourceful do tasks with the children but not those who need them”*
Individual providers (school and primary care staff)	23. Providers’ attitudes towards the program	*“Motivated preschool teachers who are happy to be involved in development”*
	24. Providers’ competence and self-efficacy	*“Great increase in skills for staff, raising equality”*
	25. Providers’ experience of workload and responsibility	*“Can be experienced by school staff as an increased workload. A change in working routines is always demanding”*
Process	26. Time for planning and executing program in school	*“It is important that time is given for planning and that it is clear how much time is required for all steps”*
	27. Communication and collaboration within school regarding process	*“That all material from Karolinska Institutet is sent out before the work at the schools takes place, which creates time for good planning in the classes”*
	28. Sustainability of program	*“That the project continues, that it is not dependent on individuals but can continue regardless of what the organisation looks like in schools and in the central administration”*

#### Step 2: Tailoring of implementation strategies

The determinants identified by stakeholders were discussed among the research team and preliminary strategies were mapped to these determinants through an internal consensus process (Strategy 17: Tailor strategies). The selection was also influenced by the work of Lyon et al. [53] who had described the most important and feasible strategies in the school context. In the second workshop in January (municipality 1) and February (municipality 2) with the same stakeholders, the implementation determinants identified in workshop 1 and the pre-selected strategies were presented. Stakeholders could then provide feedback based on their expertise and experience regarding the form and function of each strategy. No more strategies were added at this stage. After the stakeholder workshops, the scientific advisory panel was consulted to develop a final consensus on the most appropriate implementation strategies to use, which resulted in minor adjustments. The final selection of implementation strategies targeting the context-specific implementation determinants identified in Step 1 is shown in Table 2 (Basic). Based on our previous experience with the HSS program, these are regarded as a minimum to implement the HSS with reasonable fidelity in addition to first receiving formal commitment from the school principal.

**Table 2:** Implementation strategies to be deployed by all schools (bundle 1)

Implementation strategies (SISTER strategy number)	Determinants targeted	Description of the strategy	Actors	Target
Conduct local consensus discussions (23)	1. Program supported by guidelines and curriculum 5. Societal action and cooperation 8. Common goals and collaboration in school 10. Continuity in staffing and work routines 11. Leadership in school 12. Visible priority of health 23. Providers’ attitudes towards the program 27. Communication and collaboration within school 28. Sustainability of program	Introduction of the HSS to all school personnel with the help of material to create commitment and reach consensus	School principal	School personnel
Distribute educational materials (42)	8. Common goals and collaboration in school 24. Providers’ competence and self-efficacy	Order educational material at the beginnning of the new school year (manual, teachers manual, workbook for children, posters etc.) and distribute it	School health team	School personnel
		Listen to educational lectures posted on the HSS website at least once every school year whenever it fits with the schedule	School health team	School personnel
Organize school personnel implementation team meetings (32)	25. Providers’ experience of workload and responsibility 26. Time for planning and executing the program in school 27. Communication and collaboration within school regarding process	Form a health team including a teacher representative and appoint a coordinator to follow up and reflect on how to devide the practical work with the HSS, the implementation process, knowledge exchange, and how to support one another’s learning	School principal and health team	School health team
Peer-assisted learning (13)	3. Consensus on healthy lifestyle 4. Clarity of materials and instructions for use 8. Common goals and collaboration in school 23. Providers’ attitudes towards the program24. Providers’ competence and self-efficacy 26. Time for planning and executing in school 27. Communication and collaboration within school regarding process	Watch the introductory video to the classroom component, discuss a plan for implementation, how to engage parents in the home assignments	Teachers	Teachers
		Exchange of knowledge and experience in MI	School nurses	School nurses
		Internal and external meetings for inspiration, knowledge exchange and how to organise the work	School health team	School personnel
Change/alter environment (54)	6. Environmental factors 7. Local policies	Discussion regarding the possibility to make changes to support healthy lifestyle within and around the school	School health team	Community, school and after-school care
Prepare families and students to be active participants (55)	2. Involvement of parents 9. Communication and collaboration school-home 18. Family consensus on healthy lifestyle 19. Parents’ knowledge and skills 20. Socioeconomic and cultural factors, language 21. Parents’ perceived need and readiness 22. Parents’ engagement in the HSS	Announcement that the school is a health promoting school	School principal	Parents
		Introduction of the HSS at the first meeting with new parents through a film, giving the rational for the intervention, in basic Swedish and texted in other common languages	School personnel	Parents
		Information sent out in the newsletter to families of the start of the HSS. Encouragement to consult the HSS website for further information and material	School personnel	Parents

*HSS* Healthy School Start; *MI* Motivational interviewing

Six strategies will be deployed by school personnel (Table 2), supported by the material provided by the research team. At the start of the intervention and repeated yearly, the principal initiates meetings with all staff to present the HSS program and reach consensus on its execution (Strategy 23: Conduct local consensus discussions). Next, educational materials are ordered by the schools, and relevant public health lectures are made available on the project website (Strategy 42: Distribute educational materials). A health team (standard in Swedish schools) including teachers is formed and a coordinator is appointed, who will also be the contact person for the research team (Strategy 32: Organize school personnel implementation team meetings). Both teachers and school nurses engage in peer-assisted learning and make a timeplan for their respective intervention components (Strategy 13: Peer-assisted learning). If necessary, the school environment is changed to become more health promoting (Strategy 68: Change/alter environment), which may include the physical and social environment. Finally, various efforts are made to prepare families to be active participants (Strategy 58: Prepare families and students to be active participants) and consent to take part in the study through means such as information meetings, written material and a film promoting the HSS and the IMPROVE study.

Table 3 shows four additional strategies delivered to the Enhanced group only. These strategies can be characterised by regular fidelity monitoring, coaching and on-demand assistance provided by the research team to school personnel.

**Table 3:** Implementation strategies offered to group 2 schools only (bundle 2)

Implementation strategies (SISTER strategy number)	Determinants targeted	Description of the strategy	Actor	Target
Conduct educational outreach visits (38)	23. Providers’ attitudes towards the program 24. Providers’ competence and self-efficacy	Yearly presentations to schools on topics in public health of their choice and relevant to the intervention	Research team	School personnel
Promote network weaving (33)	13. Communication within and between primary health care centres 14. Time for administration in primary health care 15. Collaboration between primary care and school 16. New target groups 17. Early prevention 28. Sustainability of program	Send out yearly information letter to primary health care centres about the HSS program and the IMPROVE study. Encourage yearly meetings to establish social networks, promote information sharing, collaborative problem-solving and shared goals regarding family health	Research team	School health care and primary care
Provide ongoing consultation/coaching (44)	23. Providers’ attitudes towards the program 24. Providers’ competence and self-efficacy	Yearly audit and feedback through a written report on the fidelity score and performance of implementation strategies with coaching how to improve. E-mail sent four times per year to the school health team coordinator to offer assistance and help with problem solving	Research team	School personnel
Obtain and use student and family feedback (8)	23. Providers’ attitudes towards the program	Yearly feedback on parents’ attitude and perception of the program	Research team	School personnel

#### Step 3: Strategies performed by the research team

Implementation of the HSS intervention will start with the school year 2021/2022 and include two cohorts of children and their parents. School nurses will undergo a 2-day MI training by trainers who are members of the Motivational Interviewing Network of Trainers (MINT) and receive four supervisions (Strategy 39: Conduct ongoing training) in the spring and early fall of 2021. Lectures on relevant themes in public health will be made available on the project website, and new school staff will be trained continuously as needed. A manual will be made available for the school health team (Strategy 5: Develop a detailed implementation plan) describing the background to the HSS, the theoretical basis, goals, measures, outcomes, the time plan and strategies. Educational materials will be provided (Strategy 41: Develop educational materials) including the health information brochure, teacher’s manual, children’s workbook and other written materials to be used by the school personnel and placed on the project website. Fidelity indicators to the intervention components and instructions how to monitor them will be communicated through the manual (Strategy 7: Develop instruments to monitor and evaluate core components of new practice). A checklist to assess the quality of delivery of implementation strategies will be developed (Strategy 6: Develop and organise a quality monitoring system) and used yearly by the research team during schools visits (Strategy 9: Monitor the progress of implementation effort). An open online homepage will be made available with all information needed by different stakeholder groups and continuously updated with relevant information. School staff will be directed to this site for assistance with technical or practical questions (Strategy 11: Centralize technical assistance).

After randomisation, schools allocated to group 2 will be introduced to the strategies in bundle 2 during a meeting with the research team. Each group 2 school will be offered a check-in to inquire about their progress and assist in problem solving. It will also be possible to ask questions via the website. Finally, efforts will be made to develop or identify an organisation that will be responsible for disseminating the program and support implementation (Strategy 75: Start a dissemination organisation). This will be based on stakeholder interviews and discussions with decision-makers.

#### Step 4: Defining outcomes and data collection methods

In step 4, the outcomes were defined, and instruments to measure them were selected. Reporting of the IMPROVE study results will follow the Standards for Reporting Implementation Studies StaRI guidelines [54] and the requirements of the Consolidated Standards of Reporting Trials (CONSORT) Statement [55].

### Primary outcome

Fidelity to the four components of the HSS will be assessed through 13 indicators (Table 4) based on the dimensions adherence, dose, participant responsiveness, and quality of delivery [56, 57]. This information will be gathered through attendance logs, questionnaires answered by school staff and parents, and audio recordings of MI sessions. The primary outcome will be operationalized as an adherence score and a responsiveness score to the four intervention components. Adherence to three of the four components will be reported by parents and the nurse as yes/no. The classroom component will be reported by the teachers, and thus the score for this component will be the same for all children in the same class. The total adherence score will be from 0 to 4. Participant responsiveness measures how parents respond to and are engaged in the intervention and is measured on a scale from 1 to 5 (no appreciation to full appreciation) for each component with a total score of 4 to 20. Dose refers to exposure or the amount of the classroom component that has been delivered and received. This information will be obtained from the teachers who will use a logbook to report the proportion of teaching sessions performed, the proportion of lesson completion including length, and whether any adaptations and are made. Quality refers to the MI component. All MI sessions will be recorded and a random 10% of each nurses’ interviews will be coded for MI competence using the MITI protocol 4.2 [58] by reliable coders. Those indicators not included in the two primary outcomes will be tested separately between the Basic and Enhanced group of schools.

**Table 4:** Indicators of fidelity to the HSS components

Component	Adherence (%) (yes/no)	Dose (%)	Participant responsiveness (scale 1–5)	Quality of delivery
Brochure with health information	Parents reading the brochure (P)^a^	NA	Parents’ appreciation of the brochure (P)^b^	NA
Motivational Interview (MI)	Parents receiving MI (N)^a^	NA	Parents’ appreciation of health counselling (P)^b^	Quality of MI score coded according to MITI 4.2 (C)
Classroom component	Teachers providing the classroom component (T)^a^	Proportion of classroom lessons performed (T) Proportion of lesson completion (T)	Teachers’ perceived child involvement and engagement (T) Parent’s appreciation of the homework (P)^b^	NA
T2D test	Parents doing the test (P)^a^ Parents with a high risk that subsequently attend health care (P)	NA	Parents’ appreciation of the test (P)^b^	NA
**Total score**	Total sum score (0–4)	NA	Total sum score (4–20)	NA

Informants: *T* teacher; *C* coder; *P* parents, *N* school nurse

*NA* not assessed

^a^Included in the adherence score (primary outcome)

^b^Included in the responsiveness score (primary outcome)

### Secondary outcomes

#### Fidelity to implementation strategies

Fidelity to the implementation strategies deployed to support school personnel’s uptake and use of the intervention will be documented continuously by the research team. Once a year in May, the research team will visit each school to summarise the findings, alternatively through telephone calls. Assessing these outcomes will help to ensure that the implementation strategies are optimized and fit with end-users preferences. A checklist assessing the specific actions within each strategy will guide the assessment (Additional file 2). Each strategy will be graded as 0 = not implemented, 1 = partially implemented, 2 = fully implemented. Delivery of a strategy from the side of the research team will also be documented.

#### Acceptability, appropriateness, and feasibility

Monitoring of implementation outcomes will be done yearly with a validated and generic questionnaire developed by Weiner et al. that assesses acceptability, appropriateness, and feasibility of the HSS reported by school staff [59]. Each of these outcomes is assessed by four questions with a 5-point ordinal scale. The questionnaire will be answered individually by teachers, school nurses and principals involved in the intervention before the start and annually after that.

#### Organisational readiness to implement

This is a multi-dimensional construct that encompasses the willingness (motivation) and ability (capacity) of an entity to engage in an intervention and is considered to be a precursor of successful organisational change [22]. The results can guide internal discussions and helping identify ways to improve implementation of the HSS. School principals will answer the Leader Readiness to Implement Tool (LRIT) and school nurses and teachers will answer the Staff Readiness to Implement Tool (SRIT) adapted from the organizational readiness scale [60] by Cook et al. [61]. These tools were translated to Swedish and back-translated to English to assure correct translation. Both instruments contain 14 items on a 5-point Likert scale. Three scores will be calculated: A total readiness score, a change efficacy score, and a change commitment score.

#### Child height and weight

Child height and weight will be measured by the school nurse in pre-school class and again in year 2 including all children starting school in 2021 and 2022 (cohort 1 and 2) according to standard practice. We will use the extended international (IOTF) body mass index (kg/m^2^) cut-offs to define thinness, overweight and obesity [62]. The BMI z-score will be calculated according to a Swedish reference standard as described previously [29]. Weight and height data of all children that are routinely collected during health visits will also be obtained at the school level (de-identified, no consent needed) to be able to compare the weight status of study participants with all children at the same school.

#### Child intake of indicator foods

Dietary indicator foods (fruit, vegetables and energy-dense products), will be measured using the diet portion of a validated parent proxy questionnaire, the Eating and Physical Activity Questionnaire (EPAQ) [63], which was translated into Swedish and adapted to the Swedish context as reported previously [34]. Parents who consented to be included in the study will be asked to recall the child’s intake (servings/day) of selected foods (snacks, sweets/chocolate, ice cream, cakes/buns/cookies, fruits, vegetables, soft drinks, flavoured milk and fruit juice) during the previous day, which should be a weekday. Aggregated variables will be analysed indicating unhealthy foods (snacks, sweets/chocolate, ice-cream, cakes/buns/ cookies), healthy foods (fruit and vegetables) and unhealthy drinks (soft drink, flavoured milk and fruit juice) to analyse food patterns. The parents will be asked to answer the questionnaire at three timepoints, at baseline, at the end of the first school year, and 12 months after that.

#### Parent outcomes

Parents will receive a link by e-mail to answer online questionnaires, alternatively on paper. At baseline they will answer questions regarding their sex, educational level, country of birth, and towards the end of the school year also their responsiveness to the program components (Table 4). In total three reminders will be sent out via e-mail or text message.

##### Diabetes risk test

All parents will be encouraged to complete the T2D risk test (FINDRISC) (0–26 points) at three timepoints (at baseline and end of the first school year, and again after 12 months), in the health information brochure or on the national health care website (www.1177.se) and will receive feedback regarding their level of risk. Parents who have consented to the study will send their results to the research team using an online form.

##### Parental feeding practices

Parental feeding practices, including modelling, will be measured using the Comprehensive Feeding Practices Questionnaire (CFPQ) developed by Musher-Eizenman, covering both positive and negative practices, and validated in a middle-income US sample [64]. We will use the Swedish short version with 36 items. The questionnaire will assess the parental feeding practices of involvement, environment, food restriction for weight control, restriction for health reasons, encouragement of balance, pressure to eat, monitoring, emotion regulation, food as a reward, and modelling. The parents are asked to answer the questionnaire online at three timepoints, at baseline, at the end of the school year, and 12 months after that (Table 5).

**Table 5:** Outcome, moderators, and time of data collection

Indicator	Time of data collection			
	2021	2022	2023	2024
School characteristics	June			
Parents’ demographic data Fidelity to HSS components	October (C1)	October (C2) June (C1)	June (C2)	
Acceptability	September	June	June	June
Appropriateness	September	June	June	June
Feasibility	September	June	June	June
Organisational readiness	September	June	June	June
Fidelity to implementation strategies		May	May	May
Child height and weight	December (C1)	December (C2)	December (C1)	December (C2)
Child diet	October (C1)	June (C1) October (C2)	June (C1 + C2)	June (C2)
Parents’ T2D risk	October (C1)	June (C1) October (C2)	June (C1 + C2)	June (C2)
Parental feeding practices	October (C1)	June (C1) October (C2)	June (C1 + C2)	June (C2)

*C* cohort of children

### Stakeholder interviews

Experiences and satisfaction with the program, how it is being implemented, and how to achieve sustainability of the HSS will be assessed through in-depth interviews with a purposeful sample of nurses, school principals and representatives from the municipality management and primary health care in the latter part of the study. Semi-structured interview guides will be constructed for each target group. Interviews will be transcribed verbatim, coded and analysed using an inductive approach of content analysis [65, 66].

### Data management and statistical analysis

Data collection will be performed in a secure web platform Research Electronic Data Capture (REDCap) for building and managing online databases and surveys [67, 68]. All data will be stored electronically in password-protected folders on a secure data server with systematic back-up routines at Karolinska Institutet to avoid unauthorized access. Folders containing raw data will be stored separately from the data being used in the analysis. Access to data will be restricted to the research personnel working directly with data entry or analyses. A detailed statistical analysis plan will be developed in collaboration with an academic statistician prior to the start of data analysis.

Baseline characteristics at both school and individual level will be cross-tabulated for group 1 and 2 to check for balance and to provide an overview of the study population. Continuous and normally distributed variables will be represented as mean, standard deviation and range. Non-normally distributed variables will be represented as median and interquartile ranges, and categorical variables will be presented as frequencies and percentages in each category.

Assessment of the primary outcomes adherence score and responsiveness score after 12 and 24 months will be performed on the individual level. Between-group effects in the adherence score (0–4) will be analyzed using ordinal logistic regression. Between-group effects in the responsiveness score (4-20) will be analyzed using mixed linear regression. The following factors will be explored as potential moderators of fidelity to intervention: school size, fidelity to implementation strategies, baseline organisational readiness, parent educational level and country of birth.

In order to answer the question of whether implementation of the HSS program at scale reduces social disparities in child weight status parent’s T2D risk we will do as follows. The secondary outcomes analysed will be the change in prevalence of child overweight and obesity, children’s sex and age-adjusted BMI and parents’ risk of T2D. Dichotome outcomes will be analysed with a logistic regression model and continous outcomes with linear or quantile regression models. We will use a method able to account for the within-school and within-person clustering such as mixed models or generalised estimation equations (GEE). Parent country of birth and child sex will be explored as moderators of child and parent outcomes in addition to intervention fidelity and other school-related factors mentioned above.

Effects of implementation strategies on primary and secondary outcomes within-group will be assessed yearly relative to baseline using repeated measures analyses. All analyses will be conducted on an intention-to-treat basis. Sensitivity analysis will be carried out for primary and secondary outcomes after imputing relevant missing data, if relevant.

### Sample size and power calculation

Power based on the primary outcome “adherence score” was calculated for the sample size of 400 consenting parents in each group. Assuming that the difference in the log odds of the groups will be 0.3, 0.4 or 0.5 (maximum score 4), this corresponds to odds ratios of 1.35, 1.5 and 1.65, respectively. The power was simulated with 5000 replications and found to be 0.44, 0.66, and 0.86 for the three different scenarios. Power for the primary outcome “responsiveness score” was calculated for the same sample size as above, using the mean differences of 0.8, 1.6 and 3.2 (maximum score 20). The standard deviation was assumed to be 4, 8 and 16 for each option. With an SD of 8, the power for the three scenarios is 0.29, 0.81 and 0.99 respectively for this outcome.

### Ethical approval

This study was approved by the Swedish Ethical Review Authority (protocol number Dnr 2021–02267) on July 7, 2021. Written informed consent will be obtained from all participants in the study such as school and primary care staff and parents. Parents will be answering questionnaires, participating in recorded MI-sessions, and give permission to use routine data of their child’s height and weight.

## Discussion

Many health promotion and prevention studies have been carried out in the school context around the world aiming to improve children’s dietary habits and increase physical activity and many lessons have been learned. The HSS parental support program builds on the growing evidence base of school-based health promotion programs. Thus far, HSS has been evaluated in three cluster-randomized trials during the past 10 years in Sweden including approximately 38 schools and 1000 families (33, 34, manuscript submitted). As a universal prevention program, the HSS reaches all children and parents across the socioeconomic and multicultural spectrum. The program is fully integrated into school routines and is moderately effective in improving dietary habits and physical activity in children and in one trial BMI in children with obesity was also reduced [34]. Process evaluations have consistently shown that the program is well received among teachers, parents, children and school nurses [16, 38–40, 52]. For these reasons, the HSS program is well-positioned for implementation research that aims to develop and test strategies that facilitate its uptake and use as part of routine practice in the school setting.

The lack of evidence regarding the effect of strategies to improve implementation in the school context has been pointed out as well as the need for robust measures of implementation outcomes [69]. In the Swedish context, IMPROVE is the first study to investigate the scaling up of an evidence-based family support program in primary school. It builds on well-established frameworks and models in implementation research such as CFIR [20] and Proctor’s outcome model [46]. IMPROVE is grounded in sound implementation methods as it emphasizes a systematic approach involving first the identification of context-specific determinants in tandem with stakeholders, and then tailoring strategies to tackle the identified determinants in an effective way. Using the SISTER taxonomy [26], this research will facilitate comparisons with other implementation studies in schools, both past and present. Finally, the use of a hybrid type 3 design is advantageous when there is a high momentum and mandate for implementation and the effects of the intervention are suspected to be vulnerable to a “voltage drop” when scaling up [45].

One barrier that is specifically addressed in IMPROVE is the engagement of parents. Low parental engagement has repeatedly been shown to be a challenge in previous evaluations of the HSS [39] and in other similar programs [70]. In this study we aim to make additional efforts to engage parents by producing an attractive 5-min film explaining the purpose and content of the HSS to be watched at the first meeting in school. Also, by gaining approval of implementation of the program at the municipality level through a memorandum of understanding, school principals will be given a more prominent role as advocates of the program towards both school personnel and parents. We have also added an implementation strategy to improve the school environment, culture and climate around health, which in previous studies of the HSS has received criticisms from parents in some schools for not being health-promoting [39]. The Enhanced strategy bundle consists mainly of monitoring and coaching by the research team. There is good evidence that external facilitation will lead to higher fidelity to the intervention components [53]. The T2D component was added in a previous trial of the program to increase parent’s interest and involvement, but was unfortunately not successfully implemented. There is an ongoing discussion about the boundary between parents’ and schools’ responsibility when it comes to healthy eating and sufficient physical activity of school children [43]. The Swedish guideline for school health care does encourage schools to support parents however it does not specify how to do this [30]. Thus, the IMPROVE study can contribute to knowledge on how to increase engagement in health promotion from both parents and school personnel. From the start of developing this program the aim has been to *begin with the end in mind*, meaning that the program should be integrated into existing school routines and be scalable. This was achieved in the third wave of the program, where trained school nurses performed the MI components, as also in IMPROVE. The program is now fully compatible with school routines and in line with existing guidelines such as the curriculum, the guideline for school health care and national guidelines on the prevention of unhealthy behaviours recommending family support regarding children 6–12 years of age.

Key success strategies when scaling up school-based interventions have previously been identified by Milat et al. [71]. These are the importance of systematic use of evidence, establishing monitoring and evaluation systems, calculation of cost, active engagement of the target community through a participatory approach, tailoring to the local context, infrastructure to support implementation, strong leadership and champions, political will, well defined scale-up strategy, and strong advocacy [71]. We believe that the IMPROVE study has the potential to address most or all these key factors to successfully scale up the HSS program.

Fidelity is critical to understanding whether the failure of an intervention on client outcomes is attributable to inadequate implementation or intervention failure, or some combination thereof. In the IMPROVE study, we have developed a comprehensive measure of fidelity that will allow the exploration of the effect of implementation strategies on single intervention components and different dimensions of fidelity. Fidelity to both intervention components and implementation strategies will be measured as recommended by Slaughter [27].

What are the chances that IMPROVE will lead to the sustainment of the HSS program? Previous studies have shown that important factors for successful implementation are that the intervention has a relative advantage over other programs, that there is a tension for change, an established need in the target group, and available resources [21]. Relative advantage should be secured by the fact that no other evidence-based program in this area is available today in schools in Sweden. Tension for change among stakeholders has existed for many years given that childhood obesity and social inequalities are on the rise. The prevalence of overweight and obesity among 8–9-year-old children is around 22% and considerably higher in areas with low socioeconomic status in Sweden as elsewhere [72]. In addition, there is a higher prevalence and earlier onset of T2D in adults with low socioeconomic status [73]. However, the question still remains whether the organisation, in this case, schools and primary care, are willing to collaborate in this endeavour, which will be tested in IMPROVE. If successful, the generalisability of this universal program is believed to be high, as it has been conducted in a great number of schools already in areas with low and mixed socioeconomic status, including families from different cultural backgrounds. The intervention components are flexible and can to a large extent be adapted to family needs.

### Limitations

There is no standardised operationalisation and methodology for measuring fidelity in school-based interventions [74], which are often multi-component and multi-level interventions. In our previous studies of the HSS program, we considered MI performance and quality as a measure of fidelity, whilst observing the number of classroom sessions being performed. In IMPROVE, we have constructed a much more comprehensive fidelity measure. This measure includes 13 specific indicators covering the fidelity dimensions: adherence, dose, quality of delivery, and participant responsiveness to the four components, as well as two measures combining the four components. This will give us an opportunity to test the effect of the strategies on various aspects of fidelity to individual components and combinations thereof. However, these indicators need further validation which will be addressed during the study by e.g. observation of classroom lectures. Another limitation is that causal mechanisms on how implementation strategies work will not be studied as this was considered to be too demanding for school personnel. This could be the topic of future studies.

### WHO trial registration data set

**Table:** 

Data category	Information
Primary registry and trial identifying number	ClinicalTrials.gov ID: NCT04984421
Date of registration in primary registry	July 30, 2021
Secondary identifying numbers	None
Source(s) of monetary or material support	Swedish Research Council for Health and Working Life and Welfare (FORTE)
Primary sponsor	Swedish Research Council for Health and Working Life and Welfare (FORTE)
Secondary sponsor(s)	NA
Contact for public queries	MSc, PhD Camilla.wiklund@ki.se
Contact for scientific queries	MSc, PhD, Liselotte Schäfer Elinder, Karolinska Institutet, Stockholm, Sweden
Public title	IMPROVE
Scientific title	IMplementation and evaluation of the school-based family support PRogram A Healthy School Start to promote child health and prevent OVErweight and obesity (IMPROVE) – study protocol for a cluster-randomized trial
Country of recruitment	Sweden
Health condition(s) or problem(s) studied	Unhealthy diet, low physical activity, overweight and obesity, type 2 diabetes
Implementation intervention	All schools receive the Heathy School Start intervention. Active: Implementation strategy bundle 1 + 2 (Enhanced) Comparator: Implementation strategy bundle 1 (Basic)
Key inclusion and exclusion criteria	Inclusion: Public schools in two municipalities in the Stockholm Region. All children in pre-school class 5–7 years in these schools Exclusion: None
Study type	Parallel group cluster randomised trial
Date of first enrolment of schools	August 2021
Recruitment status	Not started
Primary outcome(s)	Fidelity to intervention (adherence and responsiveness)
Key secondary outcomes	Acceptability, appropriateness, feasibility, organizational readiness, fidelity to implementation strategies, child diet, child BMI, child weight status, parent’s risk of T2D, parental feeding practices

## Supplementary Information

**Additional file 1.** SPIRIT checklist

**Additional file 2.** Implementation strategy checklist

**Additional file 3.** Consent form

## Data Availability

Intervention materials (mostly in Swedish) are available from the project website www.ki.se/gph/enfriskskolstart. No data is yet available.

## Abbreviations

BMI: Body mass index
CFIR: Consolidated framework of implementation research
CFPQ: Comprehensive feeding practices questionnaire
EPAQ: Eating and physical activity questionnaire
ERIC: Expert recommendations for implementing change
HSS: The Healthy School Start program
LRIT: Leader readiness to implement tool
MI: Motivational interviewing
MINT: Motivational interviewing network of trainers
REDCap: Research electronic data capture
SRIT: Staff readiness to implement tool
T2D: Type 2 diabetes

## References

[1] G. B. D. Risk Factors Collaborators (2017). Global, regional, and national comparative risk assessment of 84 behavioural, environmental and occupational, and metabolic risks or clusters of risks, 1990-2016: a systematic analysis for the global burden of disease study 2016. Lancet..

[2] Commission on Ending Childhood Obesity. Report of the Commission on Ending Childhood Obesity World Health Organization; 2016.

[3] Afshin A, Forouzanfar MH, Reitsma MB, Sur P, Estep K, G. B. D. Obesity Collaborators (2017). Health effects of overweight and Obesity in 195 countries over 25 years. N Engl J Med.

[4] Elinder LS, Heinemans N, Zeebari Z, Patterson E (2014). Longitudinal changes in health behaviours and body weight among Swedish school children--associations with age, gender and parental education--the SCIP school cohort. BMC Public Health.

[5] Eustachio Colombo P, Patterson E, Elinder L, Lindroos AK. The importance of school lunches to the overall dietary intake of children in Sweden—a nationally representative study. Public Health Nutr. 2020;23(10):1705–15. 10.1017/S1368980020000099 PMC7267782 32312356

[6] Safsten E, Nyberg G, Elinder LS, Norman A, Patterson E. The intake of selected foods by six-year-old Swedish children differs according to parental education and migration status. Acta Paediatr. 2016;105(4):421-6. 10.1111/apa.13297. 10.1111/apa.13297 26663249

[7] Besharat Pour M, Bergstrom A, Bottai M, Kull I, Wickman M, Hakansson N (2014). Effect of parental migration background on childhood nutrition, physical activity, and body mass index. J Obes.

[8] Magnusson M, Sorensen TI, Olafsdottir S, Lehtinen-Jacks S, Holmen TL, Heitmann BL (2014). Social inequalities in Obesity persist in the Nordic region despite its relative affluence and equity. Curr Obes Rep.

[9] Hemmingsson E (2018). Early childhood Obesity risk factors: socioeconomic adversity, family dysfunction, offspring distress, and junk food self-medication. Curr Obes Rep.

[10] Magnusson MB, Hulthen L, Kjellgren KI (2005). Obesity, dietary pattern and physical activity among children in a suburb with a high proportion of immigrants. J Hum Nutr Diet.

[11] Hemmingsson E, Ekblom O, Kallings LV, Andersson G, Wallin P, Soderling J, et al. Prevalence and time trends of overweight, obesity and severe obesity in 447,925 Swedish adults, 1995-2017. Scandinavian Journal of Public Health. 2021;49:377–83. 10.1177/1403494820914802. 10.1177/1403494820914802 PMC8135248 32349623

[12] Waters E, de Silva-Sanigorski A, Hall BJ, Brown T, Campbell KJ, Gao Y (2011). Interventions for preventing obesity in children. Cochrane Database Syst Rev.

[13] Bleich SN, Vercammen KA, Zatz LY, Frelier JM, Ebbeling CB, Peeters A (2018). Interventions to prevent global childhood overweight and obesity: a systematic review. Lancet Diab Endocrinol.

[14] Gori D, Guaraldi F, Cinocca S, Moser G, Rucci P, Fantini MP (2017). Effectiveness of educational and lifestyle interventions to prevent paediatric obesity: systematic review and meta-analyses of randomized and non-randomized controlled trials. Obes Sci Pract.

[15] Kader M, Sundblom E, Elinder LS (2015). Effectiveness of universal parental support interventions addressing children's dietary habits, physical activity and bodyweight: a systematic review. Prev Med.

[16] Norman A, Berlin A, Sundblom E, Elinder LS, Nyberg G (2015). Stuck in a vicious circle of stress. Parental concerns and barriers to changing children's dietary and physical activity habits. Appetite..

[17] Vollmer RL, Mobley AR (2013). Parenting styles, feeding styles, and their influence on child obesogenic behaviors and body weight. A review. Appetite.

[18] McCrabb S, Lane C, Hall A, Milat A, Bauman A, Sutherland R, Yoong S, Wolfenden L (2019). Scaling-up evidence-based obesity interventions: a systematic review assessing intervention adaptations and effectiveness and quantifying the scale-up penalty. Obes Rev.

[19] Powell BJ, Beidas RS, Lewis CC, Aarons GA, McMillen JC, Proctor EK (2017). Methods to improve the selection and tailoring of implementation strategies. J Behav Health Serv Res.

[20] Damschroder LJ, Aron DC, Keith RE, Kirsh SR, Alexander JA, Lowery JC (2009). Fostering implementation of health services research findings into practice: a consolidated framework for advancing implementation science. Implement Sci.

[21] Barwik M, Dubrowski R, Damschroder L, Albers B, Shlonsky A, Mildon R (2020). Factors associated with effective implementation: Research and practical implications. Implementation Science 3.0.

[22] Scaccia JP, Cook BS, Lamont A, Wandersman A, Castellow J, Katz J, Beidas RS (2015). A practical implementation science heuristic for organizational readiness: R = MC(2). J Community Psychol.

[23] Baker R, Camosso-Stefinovic J, Gillies C, Shaw EJ, Cheater F, Flottorp S (2010). Tailored interventions to overcome identified barriers to change: effects on professional practice and health care outcomes. Cochrane Database Syst Rev.

[24] Baker R, Camosso-Stefinovic J, Gillies C, Shaw EJ, Cheater F, Flottorp S (2015). Tailored interventions to address determinants of practice. Cochrane Database Syst Rev.

[25] Powell BJ, Waltz TJ, Chinman MJ, Damschroder LJ, Smith JL, Matthieu MM, Proctor EK, Kirchner JAE (2015). A refined compilation of implementation strategies: results from the expert recommendations for implementing change (ERIC) project. Implement Sci.

[26] Cook CR, Lyon AR, Locke J, Waltz T, Powell BJ (2019). Adapting a compilation of implementation strategies to advance school-based implementation research and practice. Prev Sci.

[27] Slaughter SE, Hill JN, Snelgrove-Clarke E (2015). What is the extent and quality of documentation and reporting of fidelity to implementation strategies: a scoping review. Implement Sci.

[28] Proctor EK, Powell BJ, McMillen JC (2013). Implementation strategies: recommendations for specifying and reporting. Implement Sci.

[29] Elinder L, Patterson E, Nyberg G, Norman Å (2018). A healthy school start plus for prevention of childhood overweight and obesity in disadvantaged areas through parental support in the school setting - study protocol for a parallel group cluster randomised trial. BMC Public Health.

[30] National Board of Health and Welfare, Swedish National Agency for Education. Vägledning för elevhälsan (Guidelines for school health services). Stockholm; 2016.

[31] Quelly SB (2014). Childhood obesity prevention: a review of school nurse perceptions and practices. J Spec Pediatr Nurs.

[32] Gittelsohn J, Novotny R, Trude ACB, Butel J, Mikkelsen BE. Challenges and lessons learned from multi-level multi-component interventions to prevent and reduce childhood obesity. Int J Environ Res Public Health. 2018;16(1). 10.3390/ijerph16010030 PMC6339209 30586845

[33] Nyberg G, Sundblom E, Norman A, Bohman B, Hagberg J, Elinder LS. Effectiveness of a universal parental support programme to promote healthy dietary habits and physical activity and to prevent overweight and obesity in 6-year-old children: the healthy school start study, a cluster-randomised controlled trial. Plos One. 2015;10(2). 10.1371/journal.pone.0116876 PMC4332680 25680096

[34] Nyberg G, Norman A, Sundblom E, Zeebari Z, Elinder LS (2016). Effectiveness of a universal parental support programme to promote health behaviours and prevent overweight and obesity in 6-year-old children in disadvantaged areas, the healthy school start study II, a cluster-randomised controlled trial. Int J Behav Nutr Phys Act.

[35] Lindstrom J, Tuomilehto J (2003). The diabetes risk score: a practical tool to predict type 2 diabetes risk. Diabetes Care.

[36] Timm L, Harcke K, Karlsson I, Sidney Annerstedt K, Alvesson HM, Stattin NS, Forsberg BC, Östenson CG, Daivadanam M (2020). Early detection of type 2 diabetes in socioeconomically disadvantaged areas in Stockholm - comparing reach of community and facility-based screening. Glob Health Action.

[37] Uusitupa M, Lindstrom J, Tuomilehto J (2018). Prevention of type 2 diabetes-success story that is waiting for next steps. Eur J Clin Nutr.

[38] Bergstrom H, Sundblom E, Elinder LS, Norman A, Nyberg G (2020). Managing implementation of a parental support Programme for Obesity prevention in the school context: the importance of creating commitment in an overburdened work situation, a qualitative study. J Prim Prev.

[39] Norman A, Nyberg G, Berlin A (2019). School-based obesity prevention for busy low-income families-Organisational and personal barriers and facilitators to implementation. PLoS One.

[40] Norman A, Nyberg G, Elinder LS, Berlin A (2016). One size does not fit all-qualitative process evaluation of the healthy school start parental support programme to prevent overweight and obesity among children in disadvantaged areas in Sweden. BMC Public Health.

[41] Bergstrom H, Haggard U, Norman A, Sundblom E, Elinder LS, Nyberg G (2015). Factors influencing the implementation of a school-based parental support programme to promote health-related behaviours-interviews with teachers and parents. BMC Public Health.

[42] Howard-Drake EJ, Halliday V (2016). Exploring primary school headteachers' perspectives on the barriers and facilitators of preventing childhood obesity. J Public Health (Oxf).

[43] Clarke JL, Pallan MJ, Lancashire ER, Adab P (2017). Obesity prevention in English primary schools: headteacher perspectives. Health Promot Int.

[44] Chan AW, Tetzlaff JM, Gotzsche PC, Altman DG, Mann H, Berlin JA, Dickersin K, Hrobjartsson A, Schulz KF, Parulekar WR, Krleza-Jeric K, Laupacis A, Moher D (2013). SPIRIT 2013 explanation and elaboration: guidance for protocols of clinical trials. Bmj..

[45] Curran GM, Bauer M, Mittman B, Pyne JM, Stetler C (2012). Effectiveness-implementation hybriddesigns: combining elements of clinical effectiveness and implementation research to enhance public health impact. Med Care.

[46] Proctor E, Silmere H, Raghavan R, Hovmand P, Aarons G, Bunger A, et al. Outcomes for oimplementation research: conceptual distinctions, measurement challenges, and research agenda. Admin Pol Ment Health. 2011;38(2):65–76. 10.1007/s10488-010-0319-7. 10.1007/s10488-010-0319-7 PMC3068522 20957426

[47] National Food Agency Sweden. The Swedish Dietary Guidelines. Find your way to eat greener, not too much and be active. Sweden 2015. Avaliable from: https://www.livsmedelsverket.se/globalassets/publikationsdatabas/andra-sprak/kostraden/kostrad-eng.pdf.

[48] Miller WR, Rollnick S (2013). Motivational interviewing: Helping people change.

[49] Martins RK, McNeil DW (2009). Review of motivational interviewing in promoting health behaviors. Clin Psychol Rev.

[50] Borrelli B, Tooley EM, Scott-Sheldon LA (2015). Motivational interviewing for parent-child health interventions: a systematic review and Meta-analysis. Pediatr Dent.

[51] Saaristo T, Peltonen M, Lindstrom J, Saarikoski L, Sundvall J, Eriksson JG (2005). Cross-sectional evaluation of the Finnish diabetes risk score: a tool to identify undetected type 2 diabetes, abnormal glucose tolerance and metabolic syndrome. Diab Vasc Dis Res.

[52] Norman A, Nyberg G, Elinder LS, Berlin A (2018). Parental strategies for influencing the diet of their children - a qualitative study from disadvantaged areas. Appetite..

[53] Lyon AR, Cook CR, Locke J, Davis C, Powell BJ, Waltz TJ (2019). Importance and feasibility of an adapted set of implementation strategies in schools. J Sch Psychol.

[54] Pinnock H, Barwick M, Carpenter CR, Eldridge S, Grandes G, Griffiths CJ (2017). Standards for reporting implementation studies (StaRI) statement. BMJ..

[55] Campbell MK, Piaggio G, Elbourne DR, Altman DG, Group C (2012). Consort 2010 statement: extension to cluster randomised trials. Bmj..

[56] Allen JD, Shelton RC, Emmons KM, Linnan L, Brownson RC, Colditz GA, Proctor E (2018). Fidelity and its relationship to implementation effectiveness, adaptation and dissemination. Dissemination and implementation research in health - translating science to practice.

[57] Dusenbury L, Brannigan R, Falco M, Hansen WB (2003). A review of research on fidelity of implementation: implications for drug abuse prevention in school settings. Health Educ Res.

[58] Moyers TB, Rowell LN, Manuel JK, Ernst D, Houck JM (2016). The motivational interviewing treatment integrity code (MITI 4): rationale, preliminary reliability and validity. J Subst Abus Treat.

[59] Weiner BJ, Lewis CC, Stanick C, Powell BJ, Dorsey CN, Clary AS, Boynton MH, Halko H (2017). Psychometric assessment of three newly developed implementation outcome measures. Implement Sci.

[60] Shea CM, Jacobs SR, Esserman DA, Bruce K, Weiner BJ (2014). Organizational readiness for implementing change: a psychometric assessment of a new measure. Implement Sci.

[61] Cook C, Zhang Y, Larson M. Development and validation of two readiness to implement scales for use in the school context. Submitted manuscript 2021.

[62] Cole TJ, Lobstein T (2012). Extended international (IOTF) body mass index cut-offs for thinness, overweight and obesity. Pediatric obesity.

[63] Bennett CA, de Silva-Sanigorski AM, Nichols M, Bell AC, Swinburn BA (2009). Assessing the intake of obesity-related foods and beverages in young children: comparison of a simple population survey with 24 hr-recall. Int J Behav Nutr Phys Act.

[64] Musher-Eizenman D, Holub S (2007). Comprehensive feeding practices questionnaire: validation of a new measure of parental feeding practices. J Pediatr Psychol.

[65] Elo S, Kyngas H (2008). The qualitative content analysis process. J Adv Nurs.

[66] Krippendorff K (2013). Content analysis, an introduction to its methodology.

[67] Harris PA, Taylor R, Thielke R, Payne J, Gonzalez N, Conde JG (2009). Research electronic data capture (REDCap)--a metadata-driven methodology and workflow process for providing translational research informatics support. J Biomed Inform.

[68] Harris PA, Taylor R, Minor BL, Elliott V, Fernandez M, O'Neal L (2019). The REDCap consortium: Building an international community of software platform partners. J Biomed Inform.

[69] Wolfenden L, Nathan NK, Sutherland R, Yoong SL, Hodder RK, Wyse RJ (2017). Strategies for enhancing the implementation of school-based policies or practices targeting risk factors for chronic disease. Cochrane Database Syst Rev.

[70] Luecking CT, Vaughn AE, Burney R, Hennink-Kaminski H, Hales D, Ward DS. Fidelity and factors influencing implementation of healthy me, healthy: process evaluation of a social marketing campaign for diet and physical activity behaviors of children in childcare. Transl Behav Med. 2021;11(3):733–44. 10.1093/tbm/ibab001 PMC8034246 33538306

[71] Milat AJ, Bauman A, Redman S (2015). Narrative review of models and success factors for scaling up public health interventions. Implement Sci.

[72] Moraeus L, Lissner L, Sjoberg A (2014). Stable prevalence of obesity in Swedish schoolchildren from 2008 to 2013 but widening socio-economic gap in girls. Acta Paediatr.

[73] Agardh EE, Ahlbom A, Andersson T, Efendic S, Grill V, Hallqvist J, Ostenson CG (2004). Explanations of socioeconomic differences in excess risk of type 2 diabetes in Swedish men and women. Diabetes Care.

[74] Schaap R, Bessems K, Otten R, Kremers S, van Nassau F (2018). Measuring implementation fidelity of school-based obesity prevention programmes: a systematic review. Int J Behav Nutr Phys Act.

