# Supplementary material for: IMplementation and evaluation of the school-based family support PRogram a Healthy School Start to promote child health and prevent OVErweight and obesity (IMPROVE) – study protocol for a cluster-randomized trial
Source: BMC Public Health. 2021 Sep 6;21:1630. doi: 10.1186/s12889-021-11663-2 (PMC8419825; doi:10.1186/s12889-021-11663-2)
Supplement: Supplementary file 2 — Additional file 2. Implementation strategy checklist [file 12889_2021_11663_MOESM2_ESM.docx]

**Checklist to monitor fidelity to implementation strategies**

- All schools will be visited and asked the blue items on the checklist at the end of the school year (April each year).
- Enhanced schools will also be asked the yellow items on the checklist. Schools will be called in September 2022 and yearly thereafter and get feedback about the fidelity to intervention components based on the data collected in April. If any barrier is encountered, the research team will coach them how to resolve it.

| **Targeted strategy** | **Respondent** | **x** | **Question** | **Scoring Criteria** | **Score** |
| --- | --- | --- | --- | --- | --- |
| 2.13 Peer-assisted learning | Champion teacher in each school |  | Watch the introductory video to the classroom component | 0 = The teacher did not have any meeting, nor watch the introductory video  1 = The teacher did have meetings and watched the introductory video but there they did not assist each other  2 = The teacher had meetings and there, they assisted each other |  |
|  |  |  | Meetings between teachers to discuss a plan for implementation and how to communicate with parents about the home assignments |  |  |
|  |  |  | Assist each other regarding organisation of work with HHS program |  |  |
|  | Head nurse in each municipality |  | Exchange knowledge about the HSS program during the regular meetings with school nurses at municipality level | 0 = During the meetings the HSS program was not discussed  1 = During the meetings the HSS program was discussed  2 = During the meetings the HSS program we assisted each other |  |
| 4.23 Conduct local consensus discussions | School principal |  | Introduction of the HSS to all school personnel with the help of material provided by the RT to reach consensus | 0 = School principal has not given an introduction of the HHS  1= They have had an introduction of the HHS but not reached consensus  2 = They have had an introduction of the HHS and reached consensus |  |
| 4.32 Organize school personnel implementation team meetings | Health team coordinator in each school |  | Health team for the implementation of HSS in the school includes a teacher | 0 = Team does not include teacher  1 = Team includes teacher but does not discuss the program regularly  2 = Teem meets regularly and trains new members |  |
|  |  |  | Regular team meetings on how to divide the practical work with the HSS, the implementation process, knowledge exchange, and how to support one another’s learning |  |  |
|  |  |  |  |  |  |
| 5.42 Distribute educational materials | Health team coordinator in each school |  | Listen to educational lectures on the website at least once per year. | 0 = Health team has not distributed any educational material and haven’t listen to recorded lectures  1 = Health team has distributed some of the educational materials or have listen to recorded lectures  2 = Health team has distributed all educational materials and has listen the recorded lectures |  |
|  |  |  | Order and distribution of written material at the beginning of the new school year |  |  |
| 7.58 Prepare families and students to be active participants | Health team coordinator in each school |  | Introduction of the HSS at the first meeting with parents (introductory film) | 0 = Parents have not been introduced to the program at first meeting  1 = Parents have been introduced to the programme but no reminder sent out at start  2 = Parents have been introduced to the programme twice and have been encouraged to consult the HSS website. |  |
|  |  |  | Information sent out to parents again when HSS started |  |  |
|  |  |  | Encouragment of parents to consult the HSS website for further information and material. |  |  |
| 9.68 Change / alter environment | Health team coordinator in each school |  | Discussion about the possibility to make changes within school and around the school to support the new program | 0 = The team has not discussed the possibility to make changes within and around the school  1 = The team has discussed the possibility to make changes within and around the school  2 = The team has made changes within and around the school |  |
| 5.44 Provide ongoing consultation/coaching | Health team |  | Discussion regarding the feedback report on implementation strategies from the RT and how to improve implementation  Brief communication towards the end of the school year about the progress made with the HSS in the weekly newsletter to parents | 0= Feedback report not discussed in the health team  1= Discussion of feedback report and how to improve implementation in the health team  2= Discussion of feedback report and how to improve implementation in the health team and feedback given to parents |  |
| 4.33 Promote network weaving | Health team coordinator in each school |  | Yearly meeting with the primary health care for information sharing and collaborative problem-solving and shared goals regarding family health | 0 = The team has not met with primary care  1 = The team has planned a meeting with primary care  2 = The team has met with primary care |  |

Have there been any major changes at the school during the past school year which could have affected the program or the study?
