# Supplementary material for: IMplementation and evaluation of the school-based family support PRogram a Healthy School Start to promote child health and prevent OVErweight and obesity (IMPROVE) – study protocol for a cluster-randomized trial
Source: BMC Public Health. 2021 Sep 6;21:1630. doi: 10.1186/s12889-021-11663-2 (PMC8419825; doi:10.1186/s12889-021-11663-2)
Supplement: Supplementary file 3 — Additional file 3. Consent form [file 12889_2021_11663_MOESM3_ESM.docx]

# Consent

We agree to participate in the IMPROVE study concerning the parental support program "En Frisk Skolstart" which is carried out at our child's school.

All information about what it means to participate can be found in the attached information letter.

I have been informed and given the opportunity to ask questions and have them answered and agree to the processing of personal data.

In the case of shared custody, BOTH guardians need to sign the consent.

___________________________________________________________

Child’s name

__________________________________________Class:_____________

Child’s school and class

___________________________ ___________________________

Signature Guardian 1 Signature Guardian 2

___________________________ ___________________________

Name clarification Name clarification

___________________________ ___________________________

City and date City and date

___________________________________________________________

Email to Guardian 1

___________________________________________________________

Email to Guardian 2

___________________________________________________________

*Mailing address – NOTE only if you want paper surveys*

Submit this page today in the attached envelope, no postage needed
